# Efficacy of fingolimod is superior to injectable disease modifying therapies in second-line therapy of relapsing remitting multiple sclerosis

**DOI:** 10.1007/s00415-015-7970-6

**Published:** 2015-12-08

**Authors:** Stefan Braune, M. Lang, A. Bergmann, NeuroTransData Study Group

**Affiliations:** Neurozentrum Prien, Bernauer Str. 12, 83209 Prien, Germany

**Keywords:** Fingolimod, Disease modifying drugs, RRMS, Adherence, Relapse rate, EDSS

## Abstract

Although fingolimod is registered in Europe for treatment of relapsing-remitting multiple sclerosis (RRMS) if earlier disease modifying therapy (DMT) has failed, no data regarding its efficacy in this patient group are available. This observational cohort study of the NeuroTransData network includes German RRMS outpatients with failure of earlier therapy with injectable DMT (iDMT), therefore switching to either another iDMT (*n* = 133) or to fingolimod (*n* = 300). Statistical comparison of clinical baseline characteristics showed more severely affected patients in the fingolimod group. A propensity-score matched group comparison was performed (*n* = 99 in each group) covering more than 2-year observation time. Fingolimod showed statistically significant superior efficacy in comparison to iDMT regarding annualized relapse rate (0.21 versus 0.33 per year), time-to-relapse and likelihood of relapse (iDMT hazard ratio 1.7), proportion and likelihood of patients with EDSS progression (15.10 versus 31.00 %; iDMT hazard ratio 1.7), persistence on medication and likelihood of discontinuation (iDMT hazard ratio 3.0). Significantly more patients were free of relapse and EDSS progression with fingolimod than with their second iDMT (64.4 versus 46.5 %, *p* < 0.03). This real-life evidence in German RRMS outpatients support data from controlled clinical studies and can quantitatively support clinical decision finding processes if iDMT therapy fails in RRMS.

## Introduction

Clinical studies of fingolimod leading to registration in Europe showed superiority in clinical and MRI parameters of RRMS patients in comparison to interferon-β-1a intramuscular (TRANSFORMS [1] and placebo (FREEDOMS [2]). Based on safety concerns the market authorization by EMA decided in March 2011 (ema.europa.eu/Find medicine/Human medicines/European Public Assessment Reports) that fingolimod should be given only if RRMS patients had failed to respond to at least one other disease modifying therapy or because their disease is getting worse rapidly. Until 2011 only natalizumab was available if injectable disease modifying therapies (iDMTs, Betaferon^®^ interferon β-1b sc, Rebif^®^ interferon β-1a sc, Avonex^®^ interferon β-1a im, Copaxone^®^ glatirameracetat, Extavia^®^ interferon β-1b sc) failed with its specific benefit–risk profile associated with cases of progressive multifocal leukencephalopathy (PML) occurring since 2004. Therefore up to 79 % of RRMS patients switched within iDMTs in the US [3]. Although fingolimod offers a new treatment option since 2011 for patients failing on iDMT therapy, there is no known evidence regarding the efficacy of fingolimod in this particular clinical situation. This observational cohort study investigates the course of RRMS patients with the failure of iDMT treatment, who either switched within iDMT or to fingolimod.

## Methods

This is an observational cohort study using health data routinely collected in outpatient neurology practices throughout Germany who are members of the NeuroTransData (NTD) network. Beside demographic data clinical parameters like relapses, EDSS and medication are documented digitally in-time during clinical visits at least once within 3-month periods in all patients with MS. All neurologists are certified EDSS-rater. All participating medical staff are trained to document these data in-time in a standardized way in the web-based digital NTD data source. This data acquisition protocol is approved by the ethical committee of the Bavarian Medical Board (Bayerische Landesärztekammer, 14.06.2012). The data are pooled anonymously to form the database of the study.

This cohort analysis includes:

- RRMS patients with failure of iDMT therapy as judged by the treating neurologist and the patient between 01.01.2010 and 30.06.2015,
- who switched either to another iDMT medication or to fingolimod and,
- who had a documented observation period of a minimum of 180 days.

The decision to switch and the choice of treatment were at the discretion of the treating neurologist and the patient.

The primary outcome parameters were EDSS progression, relapse rate and adherence to medication. Progression of EDSS was defined as an increase of the EDSS score by one point if baseline EDSS was smaller than 5.5, or 0.5 points if baseline EDSS was equal or higher than 5.5.

Time-to-event analysis using Kaplan–Meyer survival curves were calculated for time-to-progression, time-to relapse and adherence including hazard ratios. Proportions of patients free of progression and/or relapses were calculated.

### Patient population

The NTD database identified 1,472 RRMS patients switching therapy. 433 of them fulfilled all inclusion criteria with 300 patients switching to fingolimod and 133 within iDMT. 59.5 % of the 1,472 patients, almost completely switching within iDMT, had to be excluded because the reason to switch was not treatment failure but others, like adverse events. Comparing baseline characteristics of clinical parameters of the fingolimod- and iDMT-cohort statistical analysis identified significant differences as shown in Table 1.

**Table 1:** Clinical Characteristics of the total cohorts of patients when switching to fingolimod or another iDMT therapy due to failure of earlier iDMT therapy

Characteristics	Fingolimod cohort *n* = 300		iDMT cohort *n* = 133		*p* value
	*n*	%	*n*	%	
Prior use of DMT before switching (*n*, % yes)	300	100.00	133	100.00	
Glatiramer acetate	66	22.00	41	30.83	
Interferon	234	78.00	92	69.17	
Years since diagnosis (*n*, %)					
Mean	8.23		5.5		<0.0001
95 % confidence interval	7.52–8.94		4.72–6.28		
Standard deviation	6.22		4.52		
Median	6		4		
EDSS baseline score when switching (*n*, %)					
Mean	2.51		1.91		0.0018
95 % confidence interval	2.32–2.69		1.62–2.20		
Standard deviation	1.49		1.3		
Median	2		2		
EDSS progression in 1 year before switching (*n*, %)	45	15.00	8	66.00	
Confirmed at least 3 months later	34	11.30	4	3.00	
Confirmed at least 6 months later	31	91.20	3	75.00	
Time since EDSS progression before switching					
Mean	106.4		175.5		0.01796
95 % confidence interval	77.96–134.84		66.15–284.85		
Standard deviation	94.67		130.79		
Median	71		158		
Relapses					
% of patients with a relapse prior to switching (*n*, %)	254	84.70	83	62.40	<0.0001
Relapse within 90 days prior to switching	131	43.70	44	33.10	0.0384
Relapse within 180 days prior to switching	185	61.70	57	42.90	0.0003
Number relapses, 1–360 days prior to switching					
0	79	26.30	61	45.90	
1	120	40.00	47	35.30	
2	60	20.00	18	13.50	
3+	41	13.70	7	5.30	
Mean ARR 1 year prior to switching	1.29		0.79		0.0002
95 % confidence interval	1.15–1.42		0.64–0.94		
Standard deviation	1.19		0.9		
Median	1		1		

In this German outpatient cohort, RRMS patients switching to fingolimod after failure of earlier iDMT therapy had a significantly longer MS duration, showed higher EDSS baseline scores, higher relapse rate and higher proportion of patient suffering EDSS progression in the previous year than patients switching within iDMT.

To enable a comparison of efficacy a propensity-score matching was performed to define comparable cohort groups. Patients were propensity score matched on EDSS score and 3 months confirmed EDSS score when starting on second therapy, number of relapses in the 360 days before starting on second therapy and years since diagnosis of RRMS. Variables considered for the propensity score model but not included were: gender, region of birth, age, and presence of relapse in the 361–720 days period before starting second therapy (Table 2).

**Table 2:** Clinical characteristics of propensity-matched cohorts of patients when switching to fingolimod or another iDMT therapy due to failure of earlier iDMT therapy

Characteristics	Fingolimod cohort (*n* = 99)		iDMT cohort (*n* = 99)		*p* value
Age when switching (years)					
Mean	39.5		40.6		
95 % confidence interval	37.6–41.3		38.6–42.7		
Standard deviation	9.3		10.2		
Median	39		40		0.4801
Gender (*n*, %)					
Male	25	25.30 %	23	23.20 %	0.7401
Female	74	74.70 %	76	76.80 %	
Days follow-up after switching (*n*, %)					
180-359	11	11.10 %	4	4.00 %	
360-719	36	36.40 %	9	9.10 %	
720+	52	52.50 %	86	86.90 %	
Mean	833.5		1242.3		
95 % confidence interval	757.1–909.9		1153.9–1330.8		
Standard deviation	383		443.4		
Median	758		1238		<0.0001
Relapses					
Proportion of patients with a relapse (*n*, %)	76	76.80 %	67	67.70 %	0.1533
Relapse in the 90 days prior to index	33	33.30 %	35	35.40 %	0.7647
Relapse in the 180 days prior to index	46	46.50 %	47	47.50 %	0.8868
Number of pre-index relapses, 1–360 days prior to index					
0	38	38.40 %	41	41.40 %	
1	41	41.40 %	38	38.40 %	
2	16	16.20 %	13	13.10 %	
3+	4	4.00 %	7	7.10 %	
Mean	0.9		0.87		0.7158
95 % confidence interval	0.70–1.10		0.68–1.05		
Standard deviation	0.98		0.93		
Median	1		1		

## Results

### Persistence on medication

**Fig. 1:**
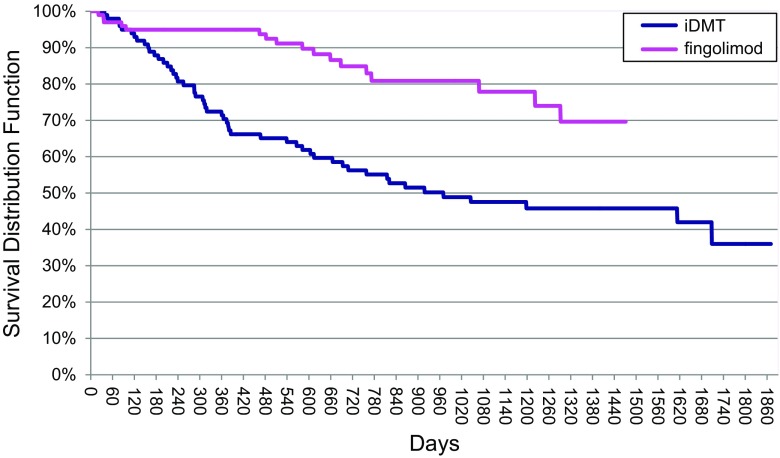
Time-to-discontinuation-of-medication analysis (Kaplan–Meier curves). Log-Rank test: Chi square 17.346 *df* 1.000, *p* value <0.0001

Persistence on medication was significantly better in the cohort with fingolimod compared to iDMT already from the very beginning of therapy with persistence after 1 year for fingolimod 95 % and iDMT 70 %, after 2 years 85 and 56 %, respectively (Fig. 1). Insufficient efficacy in about 11 times as many and side effects in about 50 % more patients with iDMT therapy than with fingolimod were causing discontinuation (Table 3).

**Table 3:** Persistence on medication in the fingolimod and iDMT matched cohorts

Characteristics	Fingolimod cohort (*n* = 99)		Idmt cohort (*n* = 99)		*p* value
Patients persistent (*n*, % yes)	82	82.80 %	47	47.50 %	<0.0001
Patients discontinued therapy (*n*, %)	12	12.10 %	36	36.40 %	<0.0001
Patient switched to another DMT (*n*, %)	5	5.10 %	16	16.20 %	0.0111
Reasons for discontinuation (*n*, % total population)					
Insufficient efficacy	2	2.00 %	22	21.80 %	
Side effects	10	9.90 %	16	15.80 %	
Pregnancy/wish for child	1	1.00 %	2	2.00 %	
Patient wish	3	3.00 %	6	5.90 %	
Other	1	1.00 %	6	5.90 %	

### Relapse rate

**Fig. 2:**
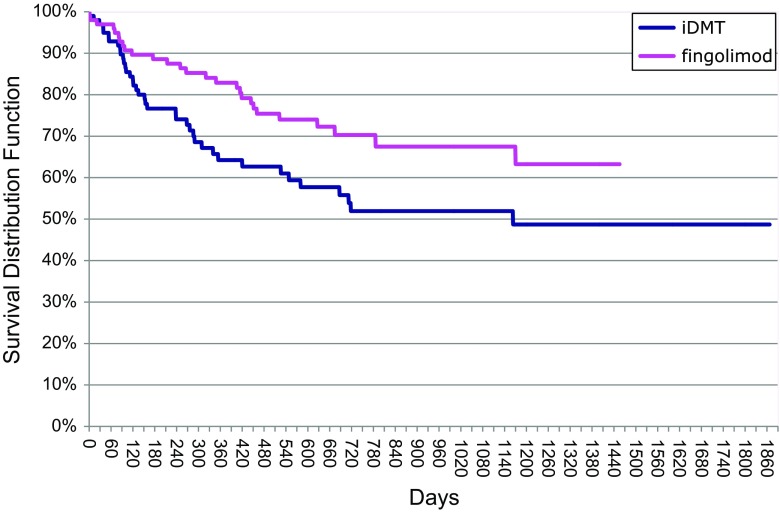
Time-to-relapse analysis (Kaplan–Meier curves). Log-Rank test: Chi square 4.982; *df* 1.000; *p* value 0.026

**Fig. 3:**
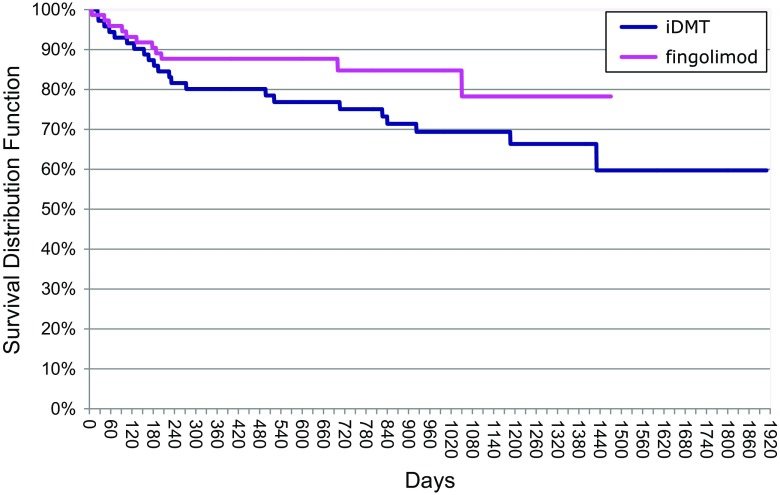
Time-to-EDSS progression analysis (Kaplan–Meier curves). Log-Rank test: Chi square 2.484; *df* 1.000; *p* value 0.115

**Fig. 4:**
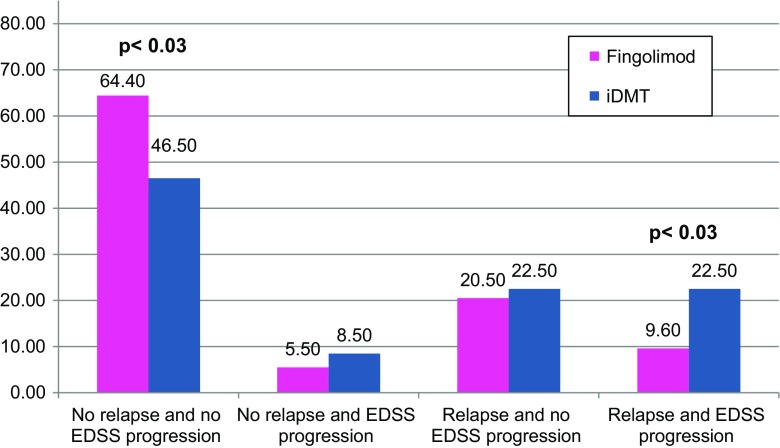
Proportion of patients with various parameters of clinical freedom of disease activity in the matched cohort treated with fingolimod or iDMT after failure of earlier iDMT therapy

Annualized relapse rate and time-to-relapse analyses were statistically significantly in favour of fingolimod (Figs. 2, 3, 4).

### EDSS progression

The number of patients showing EDSS progression during the observation time was significantly lower (*p* = 0.0231) in the fingolimod cohort (*n* = 11; 15.10 %) than in the iDMT cohort (*n* = 22; 31.00 %) (Tables 4, 5).

**Table 4:** Relapses in the fingolimod and iDMT matched cohorts

Characteristics	Fingolimod cohort (*n* = 99)		iDMT cohort (*n* = 99)		*p* value
Proportion of patients with relapse (*n*, %)	27	27.30 %	39	39.40 %	0.0704
Relapse within 90 days post switch	7	7.10 %	10	10.10 %	0.4467
Relapse within 180 days post switch	11	11.10 %	22	22.20 %	0.0359
Annualized relapse rate* (events/year)	0.21		0.33		0.0178*
95 % confidence interval	0.15–0.27		0.26–0.41		
Number of relapses					
0	72	72.70 %	60	60.60 %	
1	16	16.20 %	21	21.20 %	
2	7	7.10 %	9	9.10 %	
3+	4	4.00 %	9	9.10 %	

* Rate ratio (95 % CI) fingolimod vs iDMT: 0.63 (0.42, 0.93), *p* = 0.0178

**Table 5:** Cox proportional hazard models for matched patients with iDMT versus fingolimod therapy

Independent variables	Coefficient	Standard error	Chi square	*p* value	Hazard ratio	95 % confidence interval	
Index medication: BRACE vs fingolimod						Lower limit	Upper limit
Risk of medication discontinuation	1.113	0.281	15.665	<0.0001	3.044	1.754	5.282
Risk of relapse	0.554	0.251	4.854	0.028	1.739	1.063	2.846
Risk of EDSS progression	0.58	0.373	2.417	0.12	1.786	0.86	3.709

### Cox proportional hazard models for matched patients

Hazard ratios were higher for the cohort on iDMT compared to fingolimod, reaching statistical significance for persistence on medication and relapse.

### Freedom of clinical disease activity: NEDA 2

The proportion of patients with/without evidence of clinical MS disease activity regarding EDSS progression and/or relapse was analyzed.

During treatment with fingolimod significantly more patients remained free of relapses and EDSS progression and fewer patients suffered from relapses and EDSS progression.

## Discussion

This outpatient observational cohort study of German RRMS patients demonstrates superiority of therapy with fingolimod after failure of an earlier iDMT therapy regarding persistence on medication, relapse rate and EDSS progression compared to another medication within iDMT therapies. Significantly more patients were free of relapses and EDSS progression when treated with fingolimod compared to iDMT. This results in an impressively better persistence on fingolimod medication than on iDMT. The hazard ratios for patients switching within iDMT showed a threefold risk for discontinuation of medication and 1.7-fold risks for relapses and EDSS progression compared to fingolimod. In our matched populations the hazard ratio for discontinuation of iDMT compared to fingolimod was even higher than in previously published unmatched groups (NTD cohort hazard ratio 3.044 for iDMT versus glatirameracetat 1.75, interferon-1β 2.01 in [4]).

Overall data indicate that efficacy of fingolimod from controlled studies can be replicated in real-life regarding freedom of EDSS progression after 12 months [NTD: fingolimod 88 %, iDMT 80 %; TRANSFORM [1]: fingolimod 94 %, interferon β-1a (IFβ-1a) 92 %], and annualized relapse rate (NTD: fingolimod 0.21, iDMT 0.33; TRANSFORM [1]: fingolimod 0.16, IFβ-1a 0.33). Differences of results between this NTD cohort study and the TRANSFORM study reflect that the NTD cohort included RRMS patients with an unfavourable course during earlier iDMT therapy, while TRANSFORMS with IFβ-1a as control group included patients independent of previous course and medication.

These results can quantitatively support decision finding processes in individual RRMS patients if iDMT therapy fails. Accumulating evidence showing good cardiac safety of fingolimod even in patients with preexisting cardiac conditions [5] supports the benefit–risk considerations. Enduring persistence on medication based on clinical efficacy associated with good tolerability and safety leads to a cost-effective allocation of health system resources in favour of fingolimod compared to other DMTs [6, 7].

The impact of recent reports on PML in two patients with fingolimod with no prior exposure to immunosuppressant drugs on the benefit–risk ratio of fingolimod is under discussion as specific risk factors remain to be identified and PML seems to be associated with a number of MS immunoactive drugs.
